# Examining the impact of accreditation on a primary healthcare organization in Qatar

**DOI:** 10.1186/s12909-018-1321-0

**Published:** 2018-09-20

**Authors:** Alia Ghareeb, Hana Said, Mohamad El Zoghbi

**Affiliations:** Quality Management Department, Primary Health Care Corporation, PO Box 26555, Doha, Qatar

**Keywords:** Accreditation, Quality improvement, Primary healthcare

## Abstract

**Background:**

Although a modest body of literature exists on accreditation, little research was conducted on the impact of accreditation on primary healthcare organizations in the Middle East. This study assessed the changes resulting from the integration of Accreditation Canada International’s accreditation program in a primary healthcare organization in the State of Qatar.

**Methods:**

The study investigated how accreditation helped introduce organizational changes through promoting organizational learning as well as quality improvement initiatives. Applying a quantitative design, a structured questionnaire was used to collect data from 500 staff. The study used Spearman’s correlation coefficient to analyze the collected survey data.

**Results:**

Overall employees agreed on the positive impact of accreditation. The results showed a significantly positive correlation between staff perception of accreditation and of quality of care. The two dominant cultures at Primary Health Care Corporation were “group” (with a score of 28.61) and “hierarchical” (with a score of 26.59). The results showed a positive correlation between staff perception of accreditation and their perception of culture type whenever the culture was identified as “group”.

**Conclusions:**

This study provided much-needed insight on the possible changes that organizations might go through in relation to quality improvement and organizational learning.

## Background

Although accreditation has been widely used as a means to improve quality in secondary and tertiary care, its presence in primary care is limited [1]. This is particularly evident in the Eastern Mediterranean Region (EMR).

Accreditation systems originally developed to set standards and enhance the quality of care in acute care settings. With the expansion of primary care and the heavy emphasis placed on this sector of healthcare, accreditation organizations started to put more focus on means to improve the quality of service in primary care organizations. In Canada for instance, primary care-specific accreditation standards were developed [2].

Accreditation of primary care settings was shown to strengthen quality control and improvement [3]. Results of a study on the effectiveness of quality-improvement in primary care showed management progress in the practices that applied organizational standards [4]. A study done by Braun et al. [5] revealed that accredited primary care centers were more committed to risk management, environmental safety and quality improvement [5]. Similarly, literature review showed that accreditation of primary care organizations showed improvements in the areas of teamwork, access to care, patient safety, care processes and quality of care [2].

EMR studies revealed similar findings, a study that was conducted on primary health care centers in Lebanon emphasized on the benefits of documentation, reinforcement of quality standards and improvements in staff, and patient satisfaction [6].

Alternatively, findings of a study conducted in 2015 showed that there were gaps in the evidence on quality in primary care in the EMR region [7]. Another primary care - focused research revealed no major difference in compliance with accreditation standards between health services that went for accreditation and their counterparts. Indicators showed no such difference in efficiency as well in the areas of immunization, maternal care services, and family planning [8].

The practice of accreditation programs in primary care should be further established and assessed to be able to evaluate if participation is worthwhile or not [9]. This study aimed at filling this gap in the literature by assessing the implications of adopting accreditation in primary health care organizations. The purpose of this research was to quantitatively examine the changes resulting from the implementation of accreditation at Primary Health Care Corporation (PHCC) in the state of Qatar by studying the impact of applying Accreditation Canada International’s (ACI) standards on quality improvement and organizational learning in 21 primary health centers.

## Methods

### Setting

At the time of the research, PHCC operated through 21 health centers distributed all over the country across three main geographical regions: Central, Western, and Northern. Twelve of the existing health centers were located in Doha city (the capital and main city of the country), while the rest of the centers were located in less populated areas in other parts of the country [10, 11].

### Study design and population

This cross-sectional study was conducted in 2015 by utilizing a descriptive correlational methodology to gather data from PHCC employees who were present at the organization during the accreditation process. Data was collected from both front-line and management staff 12 months after the accreditation survey. All PHCC employees who declared to have good English language competency were the target population; that is 750 English competent employees out of close to 4000 employees. These employees worked at the 21 health centers as well as the headquarters as the organization implemented the accreditation program. Employees were managers, administrative staff, doctors, nurses, pharmacists, radiologists, technicians, support staff, and clerks. This diverse composition enabled the researcher to assess the accreditation impact from a variety of angles, from both frontline and management levels as well as from the perspectives of both healthcare providers and administrators.

### Data collection

Data was collected anonymously, without direct and indirect identifiers, using structured questionnaires that were previously used and tested for reliability. The questionnaires were adopted from Shortell’s (1995) [12] and Quinn’s and Kimberly’s (1984) [13] and amended to include the *Accreditation* and *Information about Yourself* sections [14]. The same tool was also used to measure the impact of accreditation in selected Canadian hospitals Weber (2005) [15]. This study utilized the same approach and questionnaire with minor changes to reflect primary care setting.

The survey questions were in the English language; thus, the English competent employees in PHCC were considered as eligible subjects. In order to get a representative sample, simple random sampling was used to give every subject an equal chance to be included and to reduce the risk of selection bias. The survey was sent out to the 500 randomly selected PHCC employees (out of the 750 English competent employees) and participants were given two weeks to respond. The target population staff and managers were informed through an email of the research study and were provided a link to complete the survey electronically using the *SurveyMonkey* online survey tool. The email message included all elements of an informed consent.

The questionnaires were divided into two categories (a) *Management Questionnaire*, for assessing staff perception of quality improvement, and (b) *Culture Questionnaire*, for studying the organizational learning processes.

The management questionnaire contained three main scales; *Quality of Care* (to gather information on employees’ involvement in quality improvement across seven areas or subscales: leadership, information and analysis, strategic quality planning, human resources utilization, quality management, quality results, and customer satisfaction), *Accreditation Impact* (to examine the impact of the accreditation process on dynamics of change) and *Demographics* (gender, age, occupational category, and years of experience in the corporation).

The culture questionnaire gave insight on the organizational culture in four dimensions; the organization’s character (employees perception of the organization such as being dynamic, entrepreneurial, productive, etc.…), the organization’s managers (the way managers treated employees), the organization’s cohesion (the loyalty and commitment of the organization), and the organization’s emphasis (areas related to human resources, performance, and achievements). The dependent variables were quality improvement and organizational learning. The independent variable was accreditation process.

### Statistical analysis

A correlation design using Spearmen’s correlation guided the analysis of this study. A *P*-value < 0.05 was considered significant. Data was entered and analyzed using the statistical software IBM-SPSS (Statistical Package for Social Sciences), version 21.0 (Chicago, IL, USA).

## Results

A total of 285 questionnaires were submitted electronically out of 500, for a response rate of 57%, which is relatively adequate for this type of survey [16]. A total of 253 questionnaires were included in the analysis (50.6%), and 38.8% of the submitted questionnaires (that is, 194 questionnaires) were complete.

The description of the sample is summarized in Table 1. The response rate from the health centers (48%) and the headquarters (50%) was very close indicating a roughly equal representation from both frontline and management perspectives. More females than males responded to the questionnaire (57%) which is close to the proportion of female employees in the organization, being 58% in 2015 at the time of the survey as reported by human resources department at PHCC. The majority (79%) of the respondents was under the age of 45 years, and close to two thirds of the respondents had more than ten years’ experience with the organization (63%). Out of the respondents, 15% identified themselves as occupying a managerial position. This is close to the proportion of head quarter’s employees in the organization, being 17% in 2015 at the time of the survey as reported by the human resources department at PHCC.

**Table 1:** Demographic Details

	N (253)	Percentage
Gender		
Male	109	43
Female	144	57
Age		
< =45	199	79
> 45	54	21
Years in the Organization		
< =10	180	71
> 10	73	29
Managerial Position		
Yes	37	15
No	216	85
Clinical Team		
Yes	132	52
No	121	48
Member of QMD		
Yes	41	16
No	212	84
Involved in last Accreditation		
Yes	186	74
No	67	26
Work Location		
Headquarters	122	48
Health Centers	131	52

QMD*:* Quality Management Directorate

The main clinical occupation category representing respondents was nursing (23.4%). Physicians’ rate was 8.6%. Radiology (6.6%) and dental (7%) were almost the same. For the non-clinical, the majority were administrative (21.5%), followed by coordinator level employees (12.1%). Managers were 6.6% and project managers and heads were both 8% (data not shown).

### Quality of care and accreditation impact

Data analysis of the quality of care part generated the results presented in Table 2. Interpretation of the total mean scores suggested that the areas of strengths in the quality of care variable were quality results (4.03) and leadership (4.01). The areas of weakness relative to other scales were customer satisfaction (3.79) and human resource utilization (3.67). Interpretation of findings under the seven components; leadership, information and analysis, strategic quality planning, human resources utilization, quality management, quality results, and customer satisfaction showed that employees provided considerably high ratings since all scores had a high value ranging between 3.67 and 4.03 (Table 2).

**Table 2:** Employees Perception of Quality Improvement and Accreditation

	Mean	Standard Deviation	Range
Quality Scales			
Leadership	4.01	0.69	3.64
Information and Analysis	3.94	0.66	3.00
Strategic Quality Planning	3.83	0.72	3.00
Human Resources Utilization	3.67	0.81	3.75
Quality Management	3.93	0.61	3.00
Quality Results	4.03	0.63	3.25
Customer Satisfaction	3.79	0.74	4.00
Accreditation Scales			
Overall Impact	4.17	0.57	3.00
Preparations	4.21	0.69	3.50
Recommendations	4.11	0.65	3.00
Internal Changes	4.22	0.66	3.00
Externally Oriented Changes	4.09	0.70	4.00
Valuable Tool	4.32	0.59	3.00

For the accreditation impact section, as presented in Table 2, results showed that, overall employees agreed on the positive impact of accreditation on the organization. Interpretations of the findings showed that: (a) employees were aware of and involved in the changes that were happening in preparation for accreditation (a score of 4.21). (b) They were aware of the recommendations (a score of 4.11). (c) they saw the benefit of accreditation in improving the quality of care, in the values shared in the organization, as well as in the use of internal resources (a score of 4.22). (d) They were confident in accreditation’s positive impact on addressing issues brought in by external factors like population needs and working with external stakeholders (a score of 4.09). Moreover, (e) they believed that the organization was more responsive to change due to accreditation (a score of 4.32).

### Correlational analysis between accreditation and quality of care

A correlation analysis was carried out to assess the relationship between accreditation and quality of care, as perceived by PHCC employees. As shown in Table 3, the results showed a significantly positive correlation between staff perception of accreditation and the perception of quality of care for all seven scales.

**Table 3:** Correlation between Accreditation and Quality of Care scales

		Leadership	Information and Analysis	Strategic Quality Planning	Human Resources Utilization	Quality Management	Quality Results	Customer Satisfaction
Accreditation Impact	Correlation Coefficient^a^	0.572	0.567	0.528	0.509	0.587	0.620	0.537
	*P*-value	< 0.001	< 0.001	< 0.001	< 0.001	< 0.001	< 0.001	< 0.001
		Overall Accreditation Impact	Preparations	Recommendations	Internal Changes	Externally Oriented Changes	Valuable Tool	
Quality of Care	Correlation Coefficient^a^	0.615	0.351	0.482	0.555	0.621	0.509	
	*P*-value	< 0.001	< 0.001	< 0.001	< 0.001	< 0.001	< 0.001	

^a^: Spearmen’s correlation Coefficient

The correlation analysis also showed that employees who had positive perception about accreditation for all accreditation sub- scales (preparations, recommendations, internal changes, externally-oriented changes and valuable tool) were also positive about the quality of care (p- value < 0.001 for all sections as presented in Table 3).

### Culture

Results of the culture questionnaire revealed the two dominant cultures at PHCC to be group, with a score of 28.61, and hierarchical, with a score of 26.59 as depicted in Table 4. Employees perceived PHCC to have affiliations, team work, and participation (group culture) but also had certain embedded norms and values that were associated with bureaucracy (hierarchical culture). The other two culture types, developmental and rational were 21.82 and 22.98, respectively.

**Table 4:** Employees Perception of Culture

Culture Type	Mean	Range	Standard Deviation
Group	28.61	100	14.01
Developmental	21.82	50	8.46
Hierarchical	26.59	78.75	11.93
Rational	22.98	75	10.06

### Correlational analysis between accreditation and culture

A correlation analysis was carried out to assess the relationship between accreditation and culture (see Table 5). The results showed a positive correlation between staff perception of accreditation and their perception of culture type whenever the culture was identified as group (*r* = 0.182, *p*-value = 0.011). For the hierarchical culture, there was non-significant negative correlation between the perception of accreditation and the perception of culture type (*r* = − 0.132, *p*-value = 0.067).

**Table 5:** Correlation between Accreditation Impact and Culture

		Group	Developmental	Hierarchical	Rational
Accreditation Impact	Correlation Coefficient^a^	0.182	0.093	−0.132	−0.070
	*P*-value	0.01	0.20	0.07	0.33

^a^ Spearmen’s correlation Coefficient

## Discussion

Results of this study indicated that accreditation triggered major changes in the organization at both quality improvement and organizational learning levels, thus emphasizing the importance of accreditation as a main endeavor towards improving quality in primary care. Findings also added to the body of literature that the positive impact of accreditation in relation to quality improvement and organizational learning was definite in a primary care setting, since most of the evidence found in current literature related to acute care settings.

Quality results indicated that the organization recently achieved significant improvements in quality and performance as well as in administrative areas like finance and human resources, as reported by the employees. Leadership high scores showed that the leaders of the organization had strong focus and emphasis on quality values and that quality values were integrated in the management system of the organization. As observed through data interpretation, most of the scales under quality of care had high scores, which meant that employees perceived the organization with significant progress in the areas of quality improvement and performance.

Employee involvement data indicated that whenever employees were involved in accreditation work, they had a better perception of quality in areas relating to leadership, finance, continuous quality improvement efforts, and collection of data and measurements and they were more confident about the positive changes accreditation brought during preparation, implementation, and recommendation phases.

Moreover, the correlation analysis between accreditation and quality of care sections was very strong confirming that employees perceived accreditation to be a valuable tool that triggered recent quality improvements at the organization. These results led to the conclusion that accreditation did influence the development of quality improvement practices and thus had a positive impact on quality.

Results of this study complemented what was stated in the literature about the positive impact of accreditation on quality improvement. [14, 17–21] A systematic review on healthcare accreditation reported a positive correlation between accreditation and quality programs especially during the 3 years preceding accreditation [22]. Improved compliance of healthcare organizations with the requirements of accreditation was shown to be as a tangible indication of the organizations’ effectiveness [21]. It was also reported that hospitals who were working on accreditation showed a higher compliance rate with quality standards in comparison to hospitals that were not [20]. On the other side, some discrepancies were highlighted in few studies, for example a study that was conducted in Egypt indicated that accreditation had no effect on quality, thus contradicting the findings of this research [8].

Findings of this study parallel those reported on the effect of accreditation on causing changes that influence relational and strategic changes in organizations [19] as well as on accreditation being a motivation tool that supports the quality of health services [17]. Similar to this study, research showed that accreditation was effective whenever there was strong involvement and commitment from staff [23]. In addition, healthcare professionals who were supporters of accreditation considered the process as an effective quality improvement tool that reinforced transparency and team work [24]. El Jardali et al. (2014) stated that accreditation did show improvements in the quality of health services in a study conducted in Lebanon in the primary care health centers in the country. It was found out that accreditation did have a positive impact on quality as well as on customer satisfaction. These results were in support of this research inferences on quality; however, the results contradicted with the customer satisfaction’s results since it was found in this study that accreditation did not significantly affect customer satisfaction [6]. Moreover, it was argued that improvement initiatives were only observed when organizations were preparing for the survey. The initiatives did not have a long lasting effect over time, which contradicted what is generated in this study especially that this research was conducted after one year of attainment of accreditation [25].

In this study, the predominant group culture revealed the organization as a personal place where employees had high commitment and loyalty, and managers were very caring and focusing on employees’ growth and development. The hierarchical culture was the second predominant choice and this set the organization as a very formalized and structured place governed by bureaucratic procedures and rigid policies, and characterized by permanence and efficient operational procedures. This showed that employees who were positive about accreditation perceived the culture to be of a group type, that is, they were part of a team, and they had the potential to affect quality, patient care, policy and management. They felt that they belonged to the organization. Analysis of accreditation and culture correlation, showed a positive association between staff perception of accreditation and their perception of culture type whenever the culture was identified as group. For the hierarchical culture, there was negative correlation between the perception of accreditation and the perception of culture types. This showed that employees who were positive about accreditation perceived the culture to be of a group type. Further analysis on the scores showed that employees who were involved in accreditation had a higher score for group culture in comparison to those employees who were not involved in accreditation (data not shown). These findings lead the researchers to conclude that accreditation had a positive impact on culture and, thus, on organizational learning.

Then again, correlation between accreditation and the seven scales under quality of care was positive indicating a positive impact on areas relating to leadership, information and analysis, strategic quality planning, human resources utilization, quality management, quality results and customer satisfaction. This discussion lead to the conclusion that accreditation did influence organizational learning in those areas as well.

This positive impact of accreditation on organizational learning indicated that the organization had enhanced its capabilities to produce certain desirable outcomes, and that it had the potential to institutionalize those capabilities through its employees. As a result learning and becoming accustomed to changing conditions had become monotonous, and as a result the organization developed a culture of learning [26].

Findings on organizational learning complemented what is stated in the literature about the ability of healthcare organizations that embrace certain characteristics like teamwork, communication, group affiliation, to demonstrate a broader adoption of quality improvement strategies [27]. Accreditation was also shown to create an organizational culture committed to continuous improvement of skills, teamwork, processes, product and service quality, which complemented what this research generated in relation to accreditation’s positive impact on organizational learning [28]. The study findings also lent evidence to what was stated in the literature about accreditation being a tool that aimed at both the acquisition of knowledge and the enhancement of the quality of services [18, 29, 30]. Similar to this study, it was reported that accreditation was a tool of change that fits into organizational change and the factors affecting change [31]. In the same context, accreditation was found to be a management tool that provoked change in the same sense that a quality program or a new strategic plan would [32]. The study conflicted with the findings of Sack et al. (2011) who argued that implementation of accreditation standards did not provide evidence of improvement in quality, which likewise was an absolute opposition of the findings of this study [33].

This research was distinctive because it addressed an under-researched area of accreditation in the EMR with a growing interest in studying accreditation in primary care settings. What made this study even more unique is that it addressed the primary care sector in the State of Qatar and there was no evidence on any kind of research that was conducted for the same purpose in the country.

Some limitations were identified in this study. Quality of care was assessed based on employees’ perceptions and not on measures that related to management processes or clinical outcomes. What could add more strength to the research is supplementing this set of data with measures of performance and outcomes of quality projects. There was a risk of selection bias as the population was limited to English competent employees. Another limitation was the possibility that misunderstandings might arise from the context, culture, and different interpretations of words and sentences especially that the study was conducted in English and for many of the respondents, English was a second language. Misinterpretations could be due to different understandings based on employees’ language competency and/or on previous experiences in other settings and cultures since most of employees were expatriates coming to Qatar from different countries, including Canada, the UK, South Africa, Lebanon, India, Philippines, Jordan, Syria, Egypt and other counties. An additional limitation is social desirability bias, respondents may have provided answers they considered desirable to the researchers. However, it can be safely assumed that the results should not be overstated since staff were briefed about the confidentiality and anonymity of the study. Generalizability of the study could also be considered as a limitation since the research was limited to 21 health centers in one country. Results of the study may not be applicable to other types of healthcare organizations.

A cross-sectional design was adopted in this research, in which data was collected at a single point of time. It is recommended that the research is replicated using a longitudinal study design, so that changes over time are observed. In addition, it would be very valuable to run the study in an organization prior and post to attainment of accreditation. It is also recommended that the study be conducted in both accredited and non-accredited primary care organizations and comparisons are generated to assess the differences in staff perceptions towards accreditation.

## Conclusions

High quality health care systems lead to better health outcomes and improved patient safety. Accreditation has offered a potential solution to improve the quality and learning of healthcare systems in countries all over the world including the EMR.

This study emphasizes on the positive impact of accreditation on the quality of care, as reported by PHCC employees, including areas relating to leadership, information and analysis, strategic quality planning, human resources utilization, quality management, quality results. In addition, the results supported the notion that accreditation is a drive for organizational learning.

In particular, this research gives an indication on the importance of employee involvement in accreditation and in promoting a culture that supports quality improvement and that allows employees to feel that they have a sense of belonging to the organization.

Primary care in the EMR in general and in Qatar in particular has a potential to expand services and improve outcomes. To achieve these objectives, there should be a stronger leadership focus on quality and accreditation and primary care organizations should continue to receive the necessary support and encouragement from healthcare leaders in the country. There is also a need to build the capacity of primary care organizations to allow them to embed a culture of quality improvement and thus have a continuous state of accreditation readiness for accreditation.

## Abbreviations

ACI: Accreditation Canada International
EMR: Eastern Mediterranean Region
PHCC: Primary Health Care Corporation
SPSS: Statistical Package for Social Sciences
